# Real-time simulation of the transplanted tooth using model order reduction

**DOI:** 10.3389/fbioe.2023.1201177

**Published:** 2023-06-29

**Authors:** Pierre Lahoud, Arif Badrou, Maxime Ducret, Jean-Christophe Farges, Reinhilde Jacobs, Aline Bel-Brunon, Mostafa EzEldeen, Nawfal Blal, Raphaël Richert

**Affiliations:** ^1^ OMFS-IMPATH Research Group, Department of Imaging and Pathology, Faculty of Medicine, Leuven, Belgium; ^2^ Department of Oral and Maxillofacial Surgery, University Hospitals Leuven, Leuven, Belgium; ^3^ Division of Periodontology and Oral Microbiology, Department of Oral Health Sciences, KU Leuven, Leuven, Belgium; ^4^ Laboratoire de Mécanique Des Contacts Et Structures, CNRS/INSA, Villeurbanne, France; ^5^ Laboratoire de Biologie Tissulaire Et Ingénierie Thérapeutique, UMR5305 CNRS/UCBL, Lyon, France; ^6^ Hospices Civils de Lyon, Lyon, France; ^7^ Faculty of Odontology, Lyon 1 University, Lyon, France; ^8^ Department of Dental Medicine, Karolinska Institute, Stockholm, Sweden; ^9^ Department of Oral Health Sciences, KU Leuven and Paediatric Dentistry and Special Dental Care, University Hospitals Leuven, Leuven, Belgium

**Keywords:** tooth autotransplantation, finite element analysis, real-time simulation, machine learning, model order reduction (MOR)

## Abstract

The biomechanics of transplanted teeth remain poorly understood due to a lack of models. In this context, finite element (FE) analysis has been used to evaluate the influence of occlusal morphology and root form on the biomechanical behavior of the transplanted tooth, but the construction of a FE model is extremely time-consuming. Model order reduction (MOR) techniques have been used in the medical field to reduce computing time, and the present study aimed to develop a reduced model of a transplanted tooth using the higher-order proper generalized decomposition method. The FE model of a previous study was used to learn von Mises root stress, and axial and lateral forces were used to simulate different occlusions between 75 and 175N. The error of the reduced model varied between 0.1% and 5.9% according to the subdomain, and was the highest for the highest lateral forces. The time for the FE simulation varied between 2.3 and 7.2 h. In comparison, the reduced model was built in 17s and interpolation of new results took approximately 2.10^−2^s. The use of MOR reduced the time for delivering the root stresses by a mean 5.9 h. The biomechanical behavior of a transplanted tooth simulated by FE models was accurately captured with a significant decrease of computing time. Future studies could include using jaw tracking devices for clinical use and the development of more realistic real-time simulations of tooth autotransplantation surgery.

## 1 Introduction

Replacement of permanent teeth in case of trauma appears particularly challenging in children and adolescents due to bone growth and contraindication for the use of implants. Recently, tooth autotransplantation regained interest in the dental community thanks to advancements in medical imaging. With the advent of cone beam computed tomography (CBCT), it is now possible to simulate and utilize 3D printed replicas to prepare the donor site based on the segmentation of the donor tooth. This advancement has led to reduced extra-alveolar time and improved the success rate of the surgery (EzEldeen et al., 2019). However, the risk of root fracture or root resorption of transplanted teeth is still greater than that of non-transplanted ones, and numerous biomechanical processes still remain unclear (Zhu et al., 2014; Jang et al., 2016). It was reported that excessive occlusal forces could lead to root resorption but that occlusal stimuli also facilitate the regeneration of the periodontal ligament; understanding how occlusal forces will be distributed to the root appears therefore decisive (Mine et al., 2005; Wu et al., 2019). In this context, finite element (FE) analysis has been used to evaluate the influence of occlusal morphology and root form on the biomechanical behaviour of the transplanted tooth, but only in one study (Kırmalı et al., 2022), possibly because the construction of a FE model is extremely time-consuming (Liang et al., 2018).

Numerous methods, such as deep learning-based segmentation, have been developed to automate some parts of the FE analysis (Lahoud et al., 2021). However, the computing time remains long due to large meshes and non-linearity of the periodontal ligament. In the medical field, model order reduction (MOR) techniques (Calka et al., 2021), including the most recently described higher-order proper generalised decomposition (HOPGD) method (Modesto et al., 2015; Lu et al., 2018; Badrou et al., 2023), have been used to simplify the computational complexity of biological processes and allow, for example, to simulate the blood flow with high accuracy or the displacement of the tongue. MOR techniques have yet to be employed in dentistry; herein we compared the results of a HOGPD reduced model to those of a traditional FEA approach.

## 2 Material and methods

### 2.1 Numerical method

The CBCT scan of a transplanted tooth was chosen from a previous cohort study. Detailed clinical and radiographic examinations, as well as the protocol, were previously described (EzEldeen et al., 2019; Lahoud et al., 2021). All segmented volumes were then meshed with 786,558 tetrahedral elements using the computational geometry algorithms library (CGAL) meshing library after a convergence test (Jacinto et al., 2012). A 200 μm-thick periodontal ligament was simulated using an Ogden first order hyper-elastic model around the root surface with thickness and all dental materials were supposed homogeneous and linearly elastic defined by the Young’s modulus E and by the Poisson ratio ν , for the latter (Chang et al., 2015). The strain energy function U for this law is defined as:

$$U=\frac{2\mu }{\alpha }\left({\bar{\lambda_{1}}}^{\alpha }+{\bar{\lambda_{2}}}^{\alpha }+{\bar{\lambda_{3}}}^{\alpha }-3\right)+\frac{1}{D}{\left(J-1\right)}^{2}$$ (1)

Where μ and α are material constants. D is an incompressible parameter. $\bar{\lambda_{i}}$ for i=1,2,3 have the relation $\bar{\lambda_{i}}=J^{-\frac{1}{3}}\lambda_{i}$ where $\lambda_{i}$ are the principal stretches of the left Cauchy-Green strain tensor and J is the total volume strain.

**FIGURE 1:**
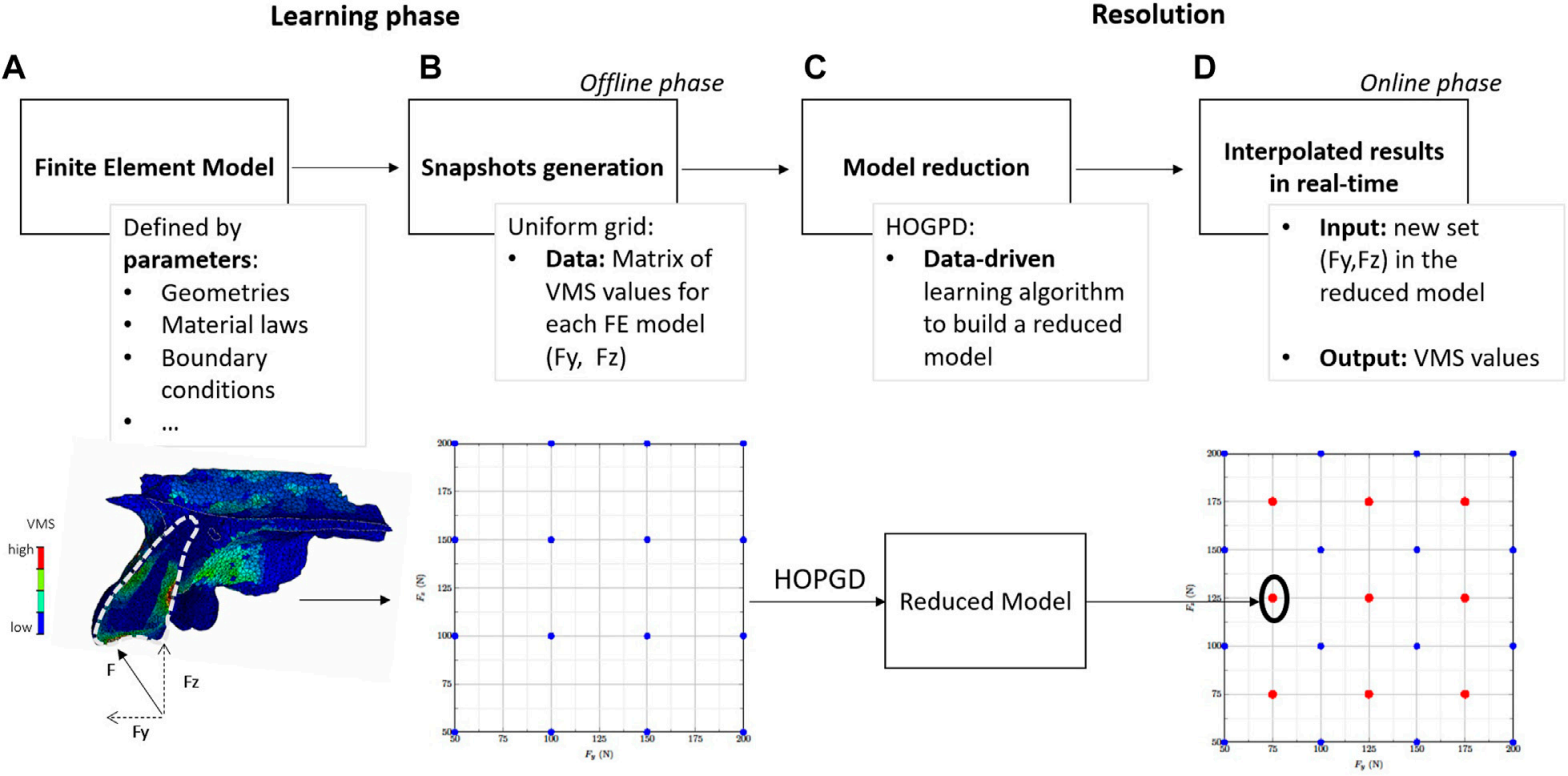
Workflow of the higher-order proper generalised decomposition (HOPGD) method. **(A)** A finite element (FE) model is created based on the anatomy of a transplanted tooth (indicated by a white dotted line). The von Mises stresses (VMS) are then calculated within the FE model under an oblique force F (represented by a black arrow), split into lateral force (Fy) and axial force (Fz). **(B)** The FE computations (blue dots) are generated in a second step using an uniform grid and VMS values for each computation are stored for data learning. In **(C)** the HOPGD algorithm is used to build a reduced model based on the previous results. **(D)** For a new set of input parameters within the parameter space (for example, the red dots in the centre of each subdomain), the reduced model can provide real-time results.

The attributed material properties were referenced from the literature (Richert et al., 2020). There was a perfect bonding between each component and the nodes of the lateral faces of the cortical bone were constrained to prevent displacement. A load was applied to the palatal face of the transplanted tooth to simulate masticatory forces. The FE analysis was conducted using the Abaqus software 6.14 (Dassault Systèmes, Vélizy-Villacoublay, France) to evaluate the von Mises root stress (VMS) of the transplanted tooth (Figure 1A). The extreme von Mises stress value was then extracted from each FE model to create a response surface.

### 2.2 Model order reduction

The general procedure follows a two-stage offline-online decomposition. In the offline stage (learning phase), snapshots (a set of results depending on space, time and model control parameters) are generated with high fidelity simulations (Figure 1B). In the online stage (in real-time), the results are interpolated with respect to the model parameters (Figure 1C).

An alternating fixed-point algorithm as proposed in (Modesto et al., 2015) can solve this minimisation problem. For a new set of parameters, the new functions were interpolated from the existing functions. In the present study, the discretisation of the parameters was conducted using uniform grids. The forces F_x_ and F_y_ were chosen as the two input parameters to simulate different occlusions ranging from an intensity from 50 to 200 N.

### 2.3 Performance and error estimation

The time for delivering root stresses calculated by FE analysis and the reduced models were compared using an Intel 2 Xeon 2.30 GHz central processing unit (CPU; Intel, Santa Clara, CA, United States).

To evaluate the accuracy of the reduced model, evaluation points are considered at the centres of the subdomains defined by the snapshot grid in the parameter space (Figure 1D). For each of these evaluation points, an additional snapshot is computed using the set of parameters at this point and the results (VMS in the present study) are stored in a reference matrix U_ref_ containing the so-called high-fidelity results. The reduced model is used to interpolate the results using the same set of parameters and the stresses obtained are in turn stored in a matrix U. For the considered point, an error *δ* is computed such that:

$$\delta =\frac{\left\|U-U_{ref}\right\|}{\left\|U_{ref}\right\|}$$ (2)

With $\left\|∙\right\|$ the L_2_ norm.

## 3 Results

**FIGURE 2:**
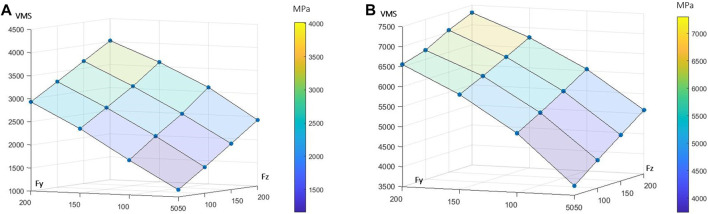
Outputs of the 16 finite element (FE) models summarised in response surfaces. Each blue node represents the extreme von Mises stress (VMS) in MPa for each of the 16 FE models. The influence of axial (F_z_) and lateral forces (F_y_) is presented for **(A)** the root and **(B)** the periodontal ligament.

For low-intensity lateral and axial loading, high stresses in the cervical part of the root and lower stresses in the centre of the canal of FE models (Figure 1A). Considering the root stresses, the extreme VMS increased linearly by 109% between the lowest and highest axial load and by 152% between the lowest and highest lateral load (Figure 2A). Considering the periodontal ligament, the extreme VMS increased linearly by 32% between the lowest and highest axial load and quadratically by 73% between the lowest and highest lateral load (Figure 2B).

**FIGURE 3:**
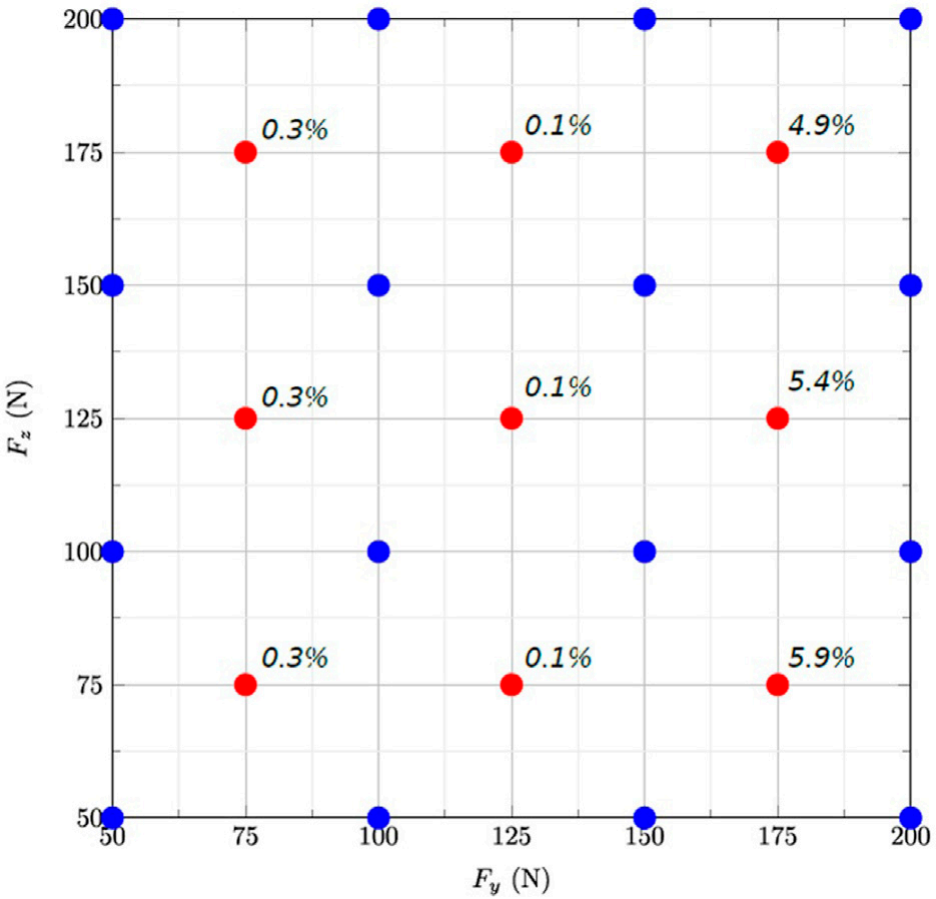
Grid representing the 2D parameter space for the two forces Fz and Fy applied on the tooth. The blue and red dots are the snapshots used to build the reduced model and those used to evaluate its accuracy using at the centres of the subdomains, respectively.

The error of the reduced model varied between 0.1% and 5.9% according to the subdomain, and was the highest for the highest lateral forces (Figure 3).

The time for the FE simulation varied between 2.3 and 7.2 h. In comparison, the reduced model was built in 17 s and interpolation of new results took approximately 2.10^−2^s (Table 1). The use of MOR reduced the time for delivering the root stresses by a mean 5.9 h.

**TABLE 1:** Comparison between data from the finite element analysis and those interpolated by the reduced model for the 9 sets of parameters at the centres of the subdomains.

F_y_/F_z_ ^a^(N)	Finite element analysis (h)	Reduced model response time (s)	Error (%)
75/75	6.6	0.024	0.3
75/125	7.0	0.021	0.3
75/175	7.1	0.020	0.3
125/75	6.9	0.021	0.1
125/125	7.0	0.022	0.1
125/175	7.2	0.019	0.1
175/75	7.1	0.019	5.9
175/125	2.3	0.020	5.4
175/175	2.6	0.020	4.9

^a^ Fy: lateral force, Fz: axial force, CPU: central processing unit; N: newton, h: hours, s: seconds.

## 4 Discussion

This proof-of-concept study demonstrated that these biomechanical aspects simulated by FE models could be captured by MOR (HOPGD) with good accuracy and with a significant decrease in computing time.

The performance of the method reported herein is in accordance with previous studies reporting a reduction of CPU time, from approximately 3 h for FEA to <1 s for reduced models (i.e., a 10^4^-fold reduction) (Lu et al., 2018) and 5 min for FEA to 10^–5^ s for reduced models (i.e., a 10^7^-fold reduction) (Badrou et al., 2023). In comparison, a proper orthogonal decomposition (POD) was used by Ng et al. for modelling the cardiac propagation and reported similar accuracy with a 10-fold reduction of computing time (Khan et al., 2020). This difference of computing times might be explained by the difference of problems to learn. It is of particular importance to note that this method also requires snapshots in an offline phase and the resolution in a reduced basis online, however one of the limitations of POD is that the enrichment of the reduced basis can quickly become expensive for high dimensional problems (Chinesta et al., 2011). By considering uniform grids, the number of snapshots is exponential. For example, a uniform grid of snapshots in a space of eight parameters with 10 values to be considered in each axis requires 10^8^ finite element snapshots. (Lu et al., 2018). Other approaches such as Proper Generalized Decomposition (PGD) based on the separation of variables, were used for haptic simulators (Quesada et al., 2018). However, the CPU time could increase by more than 100 depending on the number of modes required to construct the model (i.e., the accuracy required) (Quesada et al., 2018). Furthermore, the PGD remains an intrusive method and might therefore appear less adapted to the use of commercial software. In the present study a limited number of snapshots was chosen, but the grid could be refined for lateral forces where the error was higher. For example, a refinement strategy was recommended if the error is greater than 5% by adding snapshots along the axes of the most influential parameters in a regular grid (Lu et al., 2018). Furthermore, other approaches such as design of experiment could have been used as a first approach (Richert et al., 2022) as weakly non-linear responses were herein present. In comparison, MOR is mostly used for highly non-linear phenomena (Chinesta et al., 2011), but numerous non-linear factors were also neglected such as fatigue or contact between teeth for this first proof of concept and will be considered for future real-time simulations (Wakabayashi et al., 2008).

The root stresses were herein mostly influenced by the axis of loading, which confirms the conclusions of previous studies on the importance of occlusion (Lin et al., 2009; Hilgenfeld et al., 2019). For *in vitro*, as *in silico*, models only one occlusal situation is traditionally simulated (Richert et al., 2020), probably due to long computing times. As a consequence, our comprehension of biomechanical phenomena is limited to a particular clinical scenario (Ordinola‐Zapata et al., 2022). However, understanding occlusion is fundamental as occlusal morphology was reported to be one of the most significant variables in stress distribution in dentin and cortical bone of the transplanted tooth (Kırmalı et al., 2022). An improper occlusion can reduce the survival of the restored tooth (Bhuva et al., 2021) or impact the dental support by transmitting excessive forces (Passanezi and Sant’Ana, 2000). Conversely, it may result in premature fracture of the crown and in extreme cases root fracture. Currently, occlusal evaluation is often done using articulation papers or virtual records, which are operator-dependent and do not provide a clear understanding of force transmission in the tooth or crown (Carey et al., 2007; Kerstein and Radke, 2014; O’Carroll et al., 2019; Fraile et al., 2022). It may therefore be interesting in the future to combine the reduced model presented herein with a jaw tracking device such as Modjaw (Modjaw, Villeurbanne, France) to better understand the force transmission and adjust occlusion (Bapelle et al., 2021). Another perspective for pre-clinical students would be to use these real-time simulations with haptic simulators to better develop their surgical skills and timeliness as it was previously developed for the laparoscopic surgery (Quesada et al., 2018).

The study does have certain limitations; for instance, it is of particular importance to note that the current model was restricted to a single anatomy for a shorter learning time. Different anatomies should be tested but this appears to be complex as FE studies are currently based on a single anatomy (Richert et al., 2020). This limitation could be explained by the time needed to construct a FE model based on one tooth and future studies should evaluate how to automate this construction (Lahoud et al., 2021; Lahoud et al., 2022). In the future, it would be interesting to couple MOR with statistical shape analysis to learn also anatomic features as it has been successfully done for the aorta and the human liver (Lauzeral et al., 2019; Maquart et al., 2021). Another limitation is that the present study used simple occlusion without multiple dental contacts, as it is currently the case for most models that are limited to small deformations on relatively simple shapes and with restricted input forces (Mendizabal et al., 2020); future studies should evaluate how MOR could be adapted to learn complex occlusal schemes with multiple dental contacts. This is a major concern as computational cost could greatly increase with the complexity of the learned model.

## 5 Conclusion

This study constitutes a first proof of concept to provide real-time stress values using MOR. The biomechanical behaviour of a transplanted tooth simulated by FE models was accurately captured with a significant decrease of computing time.

## Data Availability

The raw data supporting the conclusion of this article will be made available by the authors, without undue reservation.
