# Supplementary material for: Real-time simulation of the transplanted tooth using model order reduction
Source: Front Bioeng Biotechnol. 2023 Jun 29;11:1201177. doi: 10.3389/fbioe.2023.1201177 (PMC10339382; doi:10.3389/fbioe.2023.1201177)
Supplement: Supplementary file 1 [file DataSheet1.DOCX]

Supplementary Material

Real-time simulation of the transplanted tooth using model order reduction

Pierre Lahoud, Arif Badrou, Maxime Ducret, Jean-Christophe Farges, Reinhilde Jacobs, Aline Bel-Brunon, Mostafa EzEldeen, Nawfal Blal, Raphaël Richert*

*** Correspondence:**raphael.richert@insa-lyon.fr

# Supplementary Data

We considered a function u (corresponding to the quantity of interest of the study, e.g. VMS) depending on parameters p_i_=_1,d_ that can be time, space, or control parameters of the problem. These parameters are assimilated to extra-coordinates of the solution and discretised in the parameter space [11]. The HOPGD method seeks to obtain for an approximate estimate u^n^ of u with a variable separation form such that:

$$u\left( p_{1},\ldots, p_{d} \right)\approx u^{n}\left( p_{1},\ldots,p_{d} \right)=\sum_{j=1}^{n} \prod_{i=1}^{d} F_{i}^{j}\left( p_{i} \right) (s1)$$

where n is the order of approximation and the functions $F_{i=1,d}^{j}$ are related to the j-th mode. These functions are determined by solving the minimisation problem that consists in finding u^n^ ∈ V_n_ ⊂ L^2^(Ω) minimising the cost function J such that:

$$J\left( u^{n} \right)=\min_{u^{n} \in V_{n}} \left( \frac{1}{2}\left\| u^{n}-u \right\|_{L^{2}\left( \right)}^{2} \right) (s2)$$

In this way, we have two input parameters p=4 resulting in:

$$u\left( p_{1}=\boldsymbol{x},p_{2}=t, p_{3}=F_{x},p_{4}=F_{y} \right)\approx u^{n}\left( \boldsymbol{x,}t, F_{x}F_{y} \right)=\sum_{j=1}^{n} F_{1}^{j}\left( \boldsymbol{x} \right)F_{2}^{j}\left( t \right)F_{3}^{j}\left( F_{x} \right)F_{4}^{j}\left( F_{y} \right) (s3)$$

The approximation order parameter n is inherent to the HOPGD solver and depends on the tolerated error fixed by the user
